# Addressing the health of vulnerable populations: a call for papers

**DOI:** 10.2471/BLT.16.172783

**Published:** 2016-04-01

**Authors:** Viroj Tangcharoensathien, Churnrurtai Kanchanachitra, Rebekah Thomas, James Headen Pfitzer, Paige Whitney

**Affiliations:** a International Health Policy Program, Ministry of Public Health, Nonthaburi, Thailand.; b Institute for Population and Social Research, Mahidol University, Phuthamonthon Sai 4 Road, Salaya, Phuthamonthon, 73170, Thailand.; c Gender, Equity and Human Rights, World Health Organization, Geneva, Switzerland.; d Public Health, Innovation and Intellectual Property, World Health Organization, Geneva, Switzerland.; e Essential Medicines and Health Products, World Health Organization, Geneva, Switzerland.

The end of the millennium development goals (MDGs) in 2015 marked a turning point in addressing some of the world’s most pressing and complex development challenges outlined in 2000 by the global development community. The goals, which were designed to mobilize global attention around eight priority development issues, were unprecedented in both scale and pace. Their implementation has led to significant achievements including a decline in mortality from tuberculosis and human immunodeficiency virus (HIV) infections, a decline in child and maternal mortality and improved access to safe drinking water and sanitation. These achievements show that many of the goals have been met.^1^ However, the MDGs and their focus on aggregate-level measures of progress masked the inequalities in health outcomes that existed between and within countries, regions and subgroups in a given population.^2^^,^^3^

Learning from the MDG experience, the new 2030 agenda for sustainable development has been firmly anchored in the principles of universality.^4^ Its goals are applicable to all regardless of their country’s level of development. The goals are non-discriminatory to ensure that no one is left behind.^5^ The sustainable development agenda places social justice, equity, efficacy and a people-centred approach at its core. It calls for targeted attention to the needs and rights of the most vulnerable and excluded,^4^ to ensure a life of dignity for all.^3^

While the ambitious sustainable development goals (SDGs) are welcomed, the challenge of translating them into action is significant.^5^ Effective implementation requires defining the vulnerable – those who are left behind – and giving priority to those furthest behind. Vulnerability or exclusion in its broad definition can vary significantly and may change over time.^6^

Individual factors such as sex, age, race, gender, ethnicity, displacement, disability and health status can lead to increased vulnerability of individuals and communities. These vulnerability factors often overlap and can contribute to poor health outcomes.^6^ MDG progress reports show that some population groups systematically had worse health outcome measures.^3^ The aim of targeted action focused on leaving no one behind is to recognize that vulnerable groups exist in all communities and vulnerability occurs within various social and economic contexts. 

Implementation of the SDGs requires metrics to measure inclusion and exclusion of specific population groups.^6^ There are methods in selected disciplines to measure social inclusion and exclusion, health inequality, discrimination, the cost of exclusion to societies, cost–effectiveness of addressing marginalization and promoting a universal and equitable development agenda. There are also effective interventions to tackle the causes of vulnerability in different groups and settings.

The *Bulletin of the World Health Organization* will publish a theme issue on addressing the health of vulnerable populations. For the purpose of this theme issue, the term vulnerability encompasses the effects of marginalization, exclusion and discrimination that contribute to poor health outcomes. It will focus on vulnerable groups, the drivers behind their marginalization or exclusion from wider development progress and emerging strategies to accelerate progress tailored to their needs. It will include original research articles on two challenges that are expected to be facing those implementing the* 2030 agenda for sustainable development*: how to identify those who are being left behind and how to measure and monitor progress in addressing inequality. Articles will provide experience from country-level implementation of effective interventions targeting vulnerable groups.

We welcome papers for all sections of the *Bulletin*, around four themes: (i) who, where and why vulnerable populations exist; (ii) constructs of social exclusion and the measurement of social inclusion and exclusion; (iii) interventions that reach vulnerable populations in the context of the SDGs at the micro- and macro-policy levels; (iv) factors contributing to and exacerbating vulnerabilities.

The deadline for submissions is 31 May 2016. Manuscripts should be submitted in accordance with the *Bulletin’s Guidelines for contributors* (http://submit.bwho.org), and the cover letter should mention this call for papers. All submissions will be peer reviewed.
